# Genetic dissection of a reduced seed-shattering trait acquired in rice domestication

**DOI:** 10.1270/jsbbs.23080

**Published:** 2024-08-30

**Authors:** Ryo Ishikawa

**Affiliations:** 1 Laboratory of Plant Breeding, Graduate School of Agricultural Science, Kobe University, Kobe, Hyogo 657-8501, Japan

**Keywords:** rice, domestication, seed shattering, closed panicle, *Oryza sativa*, *Oryza rufipogon*

## Abstract

Asian rice (*Oryza sativa* L.) was domesticated from wild rice (*O. rufipogon* Griff.). During rice domestication, the wild characteristic of seed-shattering behaviour was suppressed, enabling an efficient harvest with increased yield. Rice, a stable food for humans, is one of the most important crops consumed by billions of people, especially in Asian countries. With advances in molecular genetic studies, genes or loci involved in reduced seed shattering via the inhibition of abscission layer formation have been identified. The mutations alone showed no inhibitory effect on abscission layer formation in the wild rice *O. rufipogon*, but their combination enabled a stepwise change in the degree of seed shattering, which may be associated with advances in harvesting tools. In the early stages of rice domestication, the closed panicle formation and slight inhibition of the abscission layer resulted in complementary effects that increased harvesting efficiency. Furthermore, common and distinct loci were found to contribute to reduced seed shattering in groups of rice cultivars, indicating that mutations at seed-shattering loci are important information for tracing the process of rice domestication.

## Introduction

Imagine a world without rice; half of the world’s population may suffer from starvation. Rice is one of the most essential crops for nearly half of the world’s population and contributes to human development, especially in Asian countries (Khush 2005). The Asian rice *Oryza sativa* was domesticated from the wild rice, *O. rufipogon* (Khush 1997, Oka 1988). Another rice domestication event occurred in Africa, where *O. glaberrima* was domesticated from *O. barthii* (Wambugu *et al.* 2021). In both domestications, several agronomically and economically suitable traits were selected to meet with human demands, resulting in increased yields and ease of cultivation (Chen *et al.* 2019, Ishikawa *et al.* 2020).

The most striking trait for increasing edible parts was the loss of seed shattering. Wild rice exhibits seed-shattering behaviour because of the developmentally formed abscission layer between the grain and pedicel for their efficient propagation. By selecting plants that showed defects in seed-shattering behaviour, ancient gatherers may have noticed that they could grow these plants for their repeated cultivation. This trial could have been the initial step in rice domestication (Harlan *et al.* 1973). Since people have started growing these plants, additional agricultural traits have been repeatedly selected to meet with human demands and the current rice cultivars have been produced (Fuller 2007). Therefore, the loss of seed shattering may be the most critical trait serving as a springboard for domestication.

Genetic studies identifying genes or loci involved in reducing seed shattering have been extensively performed with the development of DNA markers and the genome sequencing of the cultivated rice *O. sativa* subsp. *indica* (Yu *et al.* 2002) and subsp. *japonica* (Goff *et al.* 2002, International Rice Genome Sequencing Project and Sasaki 2005). Genetic changes selected to reduce seed shattering can provide important information for clarifying the process of rice domestication. However, the exact contribution levels of genes in reduced seed shattering is currently unclear, as the trait is quantitatively regulated.

The process of rice domestication has been extensively studied using genome sequencing approaches. With advances in next-generation sequencing analyses, several phylogenetic studies have focused on the origins of rice domestication. These results are based on the sequence data of modern wild and cultivated rice, and the alternative hypotheses of several ‘origins’, such as single and multiple, are being debated (Choi *et al.* 2017, Civáň *et al.* 2015, Gross and Zhao 2014, Gutaker *et al.* 2020, Izawa *et al.* 2009, Jing *et al.* 2023, Kovach *et al.* 2007, Panaud 2009, Sang and Ge 2007a, 2007b, Wang *et al.* 2018, Wei *et al.* 2021, Zhao *et al.* 2018). Based on the sequence data of 1,083 cultivars of *O. sativa* subsp. *indica* and subsp. *japonica* as well as 446 accessions of *O. rufipogon* from Asia, the origin of rice domestication may have occurred around the middle area of the Pearl River in southern China. Subsequent introgression of *japonica* into wild rice may have produced *indica* (Huang *et al.* 2012). In contrast, Civáň *et al.* (2015) proposed three geographically separate events in Asian rice domestication based on the re-analysis of the sequence data of Huang *et al.* (2012). Other studies are ongoing to clarify the origin and process of rice domestication. The current understanding of rice domestication based on DNA sequence variations of essential domestication-related genes have been summarised in a recent review (Izawa 2022).

In this review, the current understanding of the genetic control of seed shattering in rice is reviewed. Genes or loci contributed to reduced seed shattering because of natural variations during domestication, and those identified via artificially induced mutations or reverse genetic approaches are both important for understanding the genetic regulation of seed shattering in rice. However, their mutations are carefully distinguished because the former is important for clarifying the process of domestication. Finally, future perspectives for researching seed shattering based on identification of genes or loci are discussed.

## Suppressing seed-shattering behaviour during rice domestication

Seed shattering is a typical characteristic of plant propagation. Seeds contain embryos and endosperms which contribute to reproduction and support embryo germination. Therefore, seed-shattering behaviour is evolved for the efficient propagation of flowering plants. Two seed-shattering systems are known in plants, abscission layer formation and twisting siliques which are often observed in many grain and legume crops, respectively (Dong and Wang 2015). The abscission layer is a developmentally regulated program formed in the basal part of the grain, leading to the separation of grain and pedicel. Twisting siliques are systems in which siliques with two longitudinally fused carpels can be physically shattered, and grains can be efficiently spread. Wild plants develop seed-shattering systems that are based on their growth habits.

**Fig. 1.**
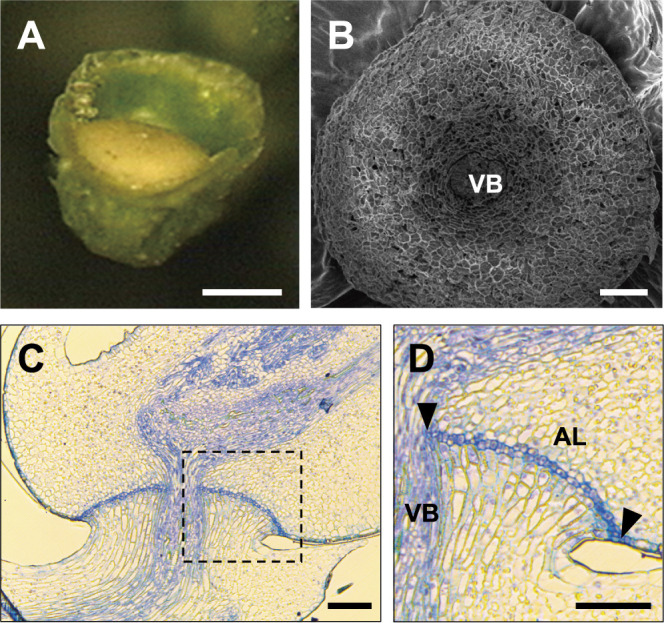
Abscission layer formation in wild rice, *Oryza rufipogon*. (A) Pedicel of rice spikelet base after detachment of grain at the seed maturation stage of wild rice *O. rufipogon* W630. Bar = 500 μm. (B) Scanning electron microscope view of abscission layer of wild rice, *O. rufipogon* W630. Bar = 50 μm. (C) Abscission layer formation investigated by vertical section at the basal part of grain and pedicel junction. (D) Magnified view of the abscission layer in dotted square of C. Black triangles indicate both edges of the abscission layer. Bars = 50 μm. VB: vascular bundle. AL: abscission layer. Bars = 50 μm.

Compared to cultivated rice, wild relatives of rice exhibit strong seed-shattering behaviour (Doi *et al.* 2008). In Asian wild rice, the annual type of *O. rufipogon*, also known as *O. nivara*, sheds seeds as soon as maturation occurs at the end of the wet season, and the parental plants generally terminate their lifecycles. The abscission layer of wild rice *O. rufipogon* is formed between the basal part of grain and pedicel (Fig. 1). Then, shattered seeds germinate in the next wet season after dormancy in the dry season. In contrast, perennial-type wild rice generally propagates through ratoons and produces fewer seeds. Therefore, seed shattering is often observed in wild rice during annual propagation.

During rice domestication, ancient people selected rice showing reduced seed-shattering behaviour, and by collecting seeds from their panicles, they may have started farming for the next harvest (Ishikawa *et al.* 2020). Currently, rice cultivars retain mature grains in their panicles, enabling to meet with efficient harvesting systems. Although seed-shattering behaviour was suppressed in cultivated rice during domestication process, a wide range of variation in the degree of seed shattering is observed in the present rice cultivars, suggesting that seed shattering is a quantitatively regulated trait (Ishii and Ishikawa 2018). In Asian cultivated rice, the widely cultivated *indica* variety generally has easy-seed-shattering behaviour, whereas some of the *japonica* variety has non-seed-shattering behaviour; both have efficient harvesting systems in their cultivation surroundings. Additionally, closing the yield gap caused by the seed shattering, especially for the *indica* variety with its easy-seed-shattering behaviour, is also an important scope for future breeding. Therefore, the genes or loci involved in the regulation of seed shattering and those associated with human selections are important.

## Genes and loci involved in the reduction of seed shattering during rice domestication

With the progress in rice genome sequencing projects, loci and genes involved in reduced seed-shattering behaviour have been identified based on genetic studies. Many quantitative trait locus (QTL) analyses for differences in the degree of seed shattering have been reported (as reviewed by Wu *et al.* 2023a), although studies confirming the effect of the QTLs on seed shattering are limited. In this section, the seed-shattering loci associated with rice domestication and those confirmed by their effects are reviewed (Table 1). First, the *sh4* locus was detected as a major QTL responsible for the degree of seed shattering in the segregating population of Asian wild rice *O. rufipogon* and *O. sativa* ssp. *indica* (Li *et al.* 2006a). Subsequently, the causal mutation at *sh4* was identified as a single nucleotide polymorphism (SNP) in the gene encoding the trihelix transcription factor (Li *et al.* 2006b, Lin *et al.* 2007). This SNP causes an amino acid substitution in the protein, that may affect its transcriptional activity. Soon after the report of *sh4*, a major locus responsible for the difference in the degree of seed shattering between cv. Kasalath and cv. Nipponbare (Npb) cultivars was identified (Konishi *et al.* 2006). The QTL was designated as *qSH1* and explained 60.0% of the total phenotypic variance. Interestingly, the causal mutation at *qSH1* is an SNP in a cis-regulatory element located upstream of the BEL transcription factor. The third locus, *qSH3*, was previously shown to be involved in the degree of seed shattering in rice (Htun *et al.* 2014, Onishi *et al.* 2007a, 2007b). Genetic dissection of *qSH3* showed that the region contained the *OsSh1* gene, a homologue of the sorghum seed-shattering gene (Lin *et al.* 2012). An SNP in the *OsSh1* gene causes an amino acid substitution which was confirmed to be associated with the degree of seed shattering based on the transgenic experiments (Ishikawa *et al.* 2022).

After the detection of *qSH3* as a locus contributing to reduced seed shattering during rice domestication, a wild introgression line (IL) carrying domesticated alleles from *japonica* Npb at *qSH1*, *sh4*, and *qSH3* was produced and designated as the IL(*qSH1*-Npb, *sh4*-Npb, *qSH3*-Npb) (Tsujimura *et al.* 2019). The wild rice used in this study is *O. rufipogon* W630, which belongs to OrI (Huang *et al.* 2012). By using wild rice, the loci contributed to the reduced seed shattering of current cultivated rice were evaluated. The seed-shattering degree of the IL(*qSH1*-Npb, *sh4*-Npb, *qSH3*-Npb) was still higher than that of Npb, implying that other loci may still be involved in further reduction in seed-shattering degree. To explore these loci, genetic analysis was conducted to the segregating population between Npb and IL(*qSH1*-Npb, *sh4*-Npb, *qSH3*-Npb). In the F_2_ segregating population with a continuous distribution of the seed-shattering degree, QTL-seq analysis was conducted (Takagi *et al.* 2013). A novel locus of *qCSS3* was found to be involved in the degree of seed-shattering, and the Npb allele was found to reduce the degree of seed shattering (Tsujimura *et al.* 2019).

Similarly, two novel seed-shattering loci, *qCSS2* and *qCSS7*, were also detected (Sugiyama *et al.* 2023). Analysis of the genotypes at three known seed-shattering loci in the *indica* rice cultivar IR36 showed that it carried domesticated alleles at *sh4* and *qSH3* and the wild allele at *qSH1* (Ishikawa *et al.* 2017). The degree of seed shattering of the IL(*sh4*-Npb, *qSH3*-Npb) carrying domesticated alleles from Npb at *sh4* and *qSH3* in the wild rice genetic background showed much lower breaking tensile strength values than those exhibited by IR36 (Sugiyama *et al.* 2023). This result implies that additional loci may still underlie the reduced seed-shattering behaviour of IR36. To explore these factors, the IL(*qSH3*-Npb, *sh4*-Npb) was crossed with IR36, and one of the resulting BC_1_F_1_ plants with the highest degree of seed shattering was selected to obtain a segregating BC_1_F_2_ population. Continuous segregation was observed for the BC_1_F_2_ population, and the causal loci associated with the degree of seed shattering were estimated using the QTL-seq method (Takagi *et al.* 2013). Two novel loci, *qCSS2* and *qCSS7*, were detected, and both IR36 alleles contributed to a reduced degree of seed shattering (Sugiyama *et al.* 2023). The effects of these two loci were further evaluated in ILs carrying IR36 alleles at *sh4*, *qSH3*, *qCSS2*, and *qCSS7* in the genetic background of wild rice (Sugiyama *et al.* 2023). The effects of *qCSS2* and *qCSS7* were detected in proportion to *sh4* and *qSH3*, and the degree of seed shattering of the IL(*sh4*-IR, *qSH3*-IR, *qCSS2*-IR, *qCSS7*-IR) was similar to that of IR36, suggesting that at least these four loci contribute to the seed-shattering degree of IR36 (Sugiyama *et al.* 2023).

The loci identified based on the difference in the degree of seed shattering between rice cultivars and wild rice were associated with selections during rice domestication. The presence of several QTLs underlying the non-seed-shattering trait in cultivated rice indicated that this trait was quantitatively regulated by multiple genes/mutations that were repeatedly selected during the domestication process. Therefore, once the causal mutations for *qCSS2*, *qCSS3*, and *qCSS7* are revealed, they will serve as valuable evidence for understanding the process of rice domestication.

## Genes controlling seed shattering identified by artificially induced mutagenesis or reverse genetic approaches

Identification of genes involved in the control of seed shattering based on analyses of artificially induced mutants or reverse genetic approaches are also important for understanding the regulation of seed shattering in rice (Table 2). However, we should focus on their genetic changes, because they are not associated with human selections during rice domestication.

Genetic analyses of several mutants have identified genes that control seed shattering. The causal gene for the easy-seed-shattering mutant *sh-h* is *OsCPL1*, which encodes the carboxy-terminal domain phosphatase-like 1 protein (Ji *et al.* 2010). Mutants with altered degree of seed shattering were screened for the chromosomal segment substitution line of cultivated rice *O. sativa* *indica*, which carries the whole chromosome 4 of the wild rice *O. rufipogon* (Zhou *et al.* 2012). The causal gene for the shattering-resistant mutant, *shat1* (*shattering abortion 1*), was found to be the APETALA2 transcription factor that controls the specification of the abscission zone. Similarly, based on the analysis of the mutant, the *SSH1* (*Suppression of Shattering 1*) gene was found to be *SUPERNUMERARY BRACT* (*SNB*), encoding a plant-specific APETALA2-like transcription factor (Jiang *et al.* 2019). The easy-seed-shattering cultivar was subjected to gamma-ray irradiation to breed a shattering-resistant cultivar (Li *et al.* 2019). The causal locus responsible for the change in the degree of seed shattering was found to encode the microRNA (*osa-mir172d*).

Previously, *Sh1* encoding a YABBY transcription factor was identified by analysing the reduced seed-shattering behaviour of sorghum (Lin *et al.* 2012). The orthologous gene *OsSh1* was also deleted in the non-shattering mutant SR-5 obtained by artificial gamma-ray irradiation. Similarly, a mutation in another non-shattering cultivar, developed via gamma-ray irradiation, was found in *OsSh1* (Li *et al.* 2020). These *OsSh1* mutations were artificially induced and were distinct form the SNP, causing an amino acid substitution in *OsSh1*, which was then selected to reduce the degree of seed shattering during rice domestication (Ishikawa *et al.* 2022; Table 1).

A previous study showed that an SNP at *qSH1* is responsible for controlling the expression of the BEL1-like homeobox gene (Konishi *et al.* 2006). Among the 17 homologous genes, *SH5* was the closest to the *qSH1*-controlling gene, and is involved in the control of seed shattering (Yoon *et al.* 2014). BELL proteins are known to form complexes with KNOX proteins. Among the rice KNOX proteins, *OSH15* was found to regulate seed shattering, as a T-DNA insertion mutant of *OSH15* which reduced the reduced degree of seed shattering (Yoon *et al.* 2017). The interaction between OSH15 and BELL-type homeobox proteins, including SH5, directly inhibited the expression of lignin biosynthesis genes, which are essential for abscission layer formation. Recently, gibberellin signalling was found to be important for regulating lignin biosynthesis as it modulates seed-shattering genes. SLR1, a gibberellin signalling repressors, interacts with SNB, OSH15, and BELL protein encoded by *qSH1* (Wu *et al.* 2023b).

The natural variations of these genes may have been selected, to reduce the degree of seed shattering during rice domestication, as some may have a surrounding region with a selective signature. However, the artificially induced mutations are unlikely to be associated with domestication. Therefore, their roles in rice domestication must be carefully analysed.

## Genes controlling seed shattering based on the studies of African cultivated rice, *O. glaberrima*

Besides Asian cultivated rice, another cultivated rice independently domesticated in Africa is known as *O. glaberrima* Steud, which was domesticated from the African wild rice, *O. barthii*. The primary origin of *O. glaberrima* has been proposed to be in the Niger River Delta (Portéres 1962). Similar traits were also selected during domestication in *O. glaberrima* as compared to the Asian rice, *O. sativa*. Loss of seed shattering is also an important domestication-related trait in African rice. Two loci were previously identified as being involved in reducing seed shattering during the domestication of *O. glaberrima* (Table 3). *Sh3* was originally identified as a locus controlling seed shattering of wild rice species (Eiguchi and Sano 1990). Further analysis of the *Sh3* locus in *O. glaberrima* showed that causal mutation was found in *sh4*, a gene referred to by Li *et al.* (2006b) that is involved in reduced seed-shattering behaviour in Asian cultivated rice, *O. sativa*. Mutation of the *sh4* gene found in *O. glaberrima* is an SNP that leads to a premature stop codon, and also affects grain size (Win *et al.* 2017). Similarly, a genetic study of grain size differences between *O. barthii* W1411 and *O. glaberrima* detected *GL4*, a QTL in which the *O. barthii* allele contributes to longer and larger grain sizes (Wu *et al.* 2017). The authors also reported that *GL4* is an allele of *sh4* thereby affecting the degree of seed shattering. Because the *O. glaberrima* allele of *sh4* causes a smaller grain size and a reduction in seed-shattering behaviour, human selection is likely targeted to reduce seed-shattering behaviour rather than grain size. The mutation in the *sh4* gene was distinct between *O. glaberrima* and *O. sativa*, suggesting that the *sh4* gene was targeted to reduce the seed-shattering behaviour of the both two cultivated rice.

The genome sequencing analysis of *O. glaberrima* and population genetic studies have identified a large deletion in the region containing *OsSh1*, suggesting that this deletion mutation may have been selected during the domestication of *O. glaberrima* (Wang *et al.* 2014). Later, the locus was also studied as *SH3*, a major QTL controlling the degree of seed shattering between *O. graberrima* (accession no. IRGC104165 with the wild allele at *SH4*), and *O. barthii* W1411 (Lv *et al.* 2018). The QTL was further analysed, and causal mutation was identified as a large deletion containing *OsSh1*, confirming that the locus contributed to the reduced degree of seed shattering. The causal mutation of *OsSh1* in Asian cultivated rice was found to be an SNP (Ishikawa *et al.* 2022), however, the mutation in the African cultivated rice differs depending on the cultivar. These selections at the same loci but with distinct mutations indicate that convergent but independent processes underlie the domestication of Asian and African rice (Purugganan 2014).

In addition, using the genomic dataset of 163 *O. glaberrima* and 83 *O. barthii* accessions collected in the Sahel zone and East Africa, a selective signature was observed at the *SH5* region, a locus previously reported to be involved in the regulation of seed shattering in *O. sativa* (Cubry *et al.* 2018). A deletion is found to be fixed in *O. glaberrima* cultivars, suggesting that the mutation was probably involved in reducing seed shattering during African rice domestication; however, the effect of the mutation on the degree of shattering remains to be confirmed. These loci detected in *O. glaberrima* are likely involved in reducing seed shattering, but the extent to which these processes sequentially or distinctly contribute to the reduction in seed shattering remains to be elucidated, necessitating the use of a panel of *O. glaberrima* cultivars.

Recently, *SH11*, a novel seed-shattering gene encoding a MYB transcription factor, was detected using *O. glaberrima*. Among a set of ILs between *O. glaberrima* WK21 (accession No. IRGC104038) and *O. sativa* Tauchung 65 (T65), an IL harbouring a chromosomal segment of WK21 in a T65 background, showed reduced seed-shattering behaviour (Ning *et al.* 2023). Genetic mapping and transformation analyses revealed that WK21 and T65 encode functional and nonfunctional alleles, respectively. Sequencing analysis showed that the two polymorphisms were likely causal mutations between WK21 and T65 (Ning *et al.* 2023). Further genetic analysis revealed that *SH11* downregulated the expression of lignin biosynthesis genes. However, involvement of *SH11* gene in the domestication of *O. glaberrima* and *O. sativa* requires further elucidation. The currently identified genes or loci contributing to the reduced seed-shattering behaviour in *O. glaberrima* are relatively fewer than those detected in studies on *O. sativa*, but some of them commonly contribute to reducing the degree of seed shattering of *O. glaberrima*.

## Role of panicle shape on reducing seed shattering in early rice domestication

Several loci involved in reducing the degree of seed shattering by human selections have been detected in the Asian cultivated rice, *O. sativa* (Table 1). Since the *qSH1* mutation is only observed in some *japonica* rice cultivars, the locus contributed to reduced seed shattering, specifically in *japonica* rice and at relatively later stages of rice domestication. Mutations at *sh4* and *qSH3* are both conserved in *indica* and *japonica* rice cultivars, suggesting these two loci may have played an important role in the initial suppression of seed shattering when wild rice was first targeted for selection (Ishikawa *et al.* 2022). The effects of a single mutation at the two loci on seed shattering were evaluated using ILs in wild rice genetic background: namely IL(*sh4*-Npb) and IL(*qSH3*-Npb). No difference was detected in abscission layer formation between the two ILs and wild rice, suggesting that their single mutations were not sufficient to confer phenotypic changes on seed-shattering behaviour in wild rice (Ishikawa *et al.* 2022). Then the two mutations were evaluated together in the IL in wild rice genetic background. The IL(*sh4*-Npb, *qSH3*-Npb) showed a slight inhibition of the abscission layer around vascular bundles (Inoue *et al.* 2015, Ishikawa *et al.* 2022). The partial connection of the basal part of the grain to the pedicel in the abscission layer is thought to be an important step in the initial loss of seed shattering. However, when the IL(*sh4*-Npb, *qSH3*-Npb) was grown in the field, their seeds were also shattered like wild rice. Under field conditions, such a slight inhibition of the abscission layer is not sufficient to sustain seeds in the panicle, as the wild rice has an open panicle structure that promotes seed shattering when a little force is applied to the grain with awns. Closed panicles were also selected during rice domestication, as they retain seeds in the panicle and promote self-fertilisation owing to their long awns on lemmas (Ishii *et al.* 2013). *SPR3* was found to control panicle shape as the locus controlling the expression of the downstream gene *OsLG1*. Moreover, the *SPR3* region exhibited a selective signature in both *indica* and *japonica* rice. Therefore, the closed panicle is a trait that is selected for relatively early in rice domestication (Ishii *et al.* 2013, Zhu *et al.* 2013).

**Fig. 2.**
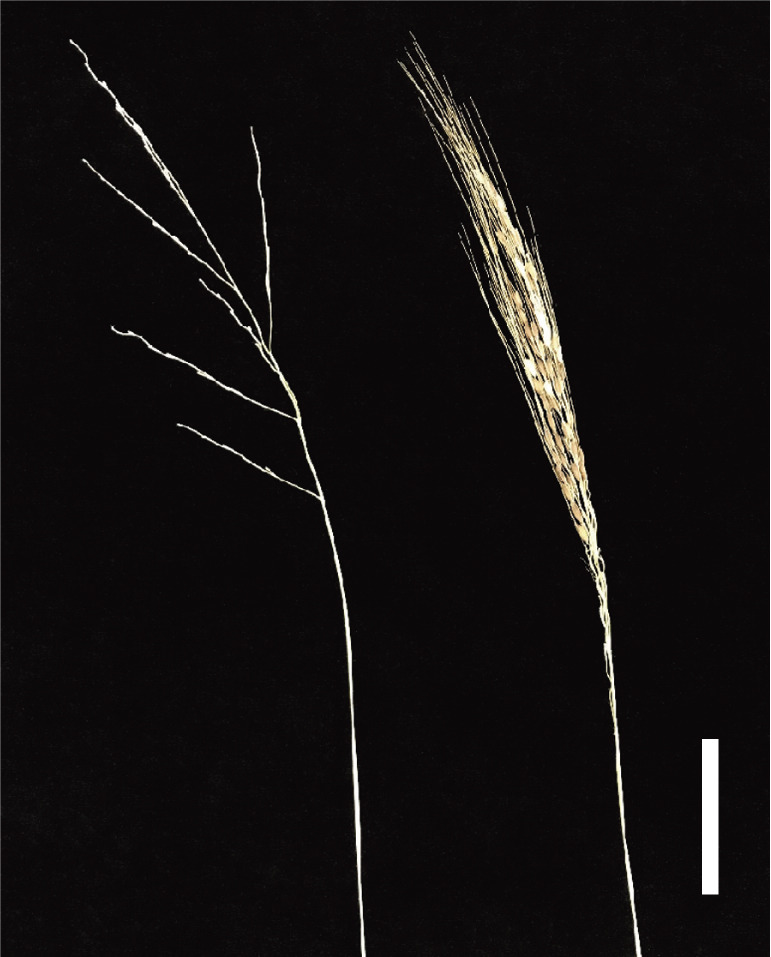
Comparison of panicle morphologies between wild rice *Oryza rufipogon* and an introgression line (IL) of wild rice carrying chromosomal segments of *O. sativa* Nipponbare covering *sh4*, *qSH3*, and *SPR3* regions. (left) *O. rufipogon* W630, (right) IL(*sh4*-Npb, *qSH3*-Npb, *SPR3*-Npb). Most of the grains were left in the panicle in the IL. Bar = 5 cm.

To better understand the initial stage of reducing seed shattering in rice domestication, the interaction between panicle shape and reduction in seed shattering was investigated in wild rice. ILs carrying a combination of Npb chromosomal segments harbouring *qSH3*, *sh4*, and *SPR3* have also been produced (Ishikawa *et al.* 2022). Seven ILs in combinations with the three loci, as well as wild rice, were grown in paddy fields, and their seed-gathering rates were estimated. ILs with one or two domesticated alleles showed low harvesting efficiency, whereas IL with three domesticated alleles showed the highest efficiency, showing that closed panicles had a promoting effect on the reduction in seed shattering when slight inhibition of the abscission layer was achieved by *qSH3* and *sh4* interaction. Wild rice with visible seeds remaining in the panicle may have been selected by ancient gatherers at the start of rice domestication (Fig. 2).

The interaction of the closed panicle and the inhibition of the abscission layer were further studied based on structural mechanics. Inhibition of the abscission layer occurs around vascular bundles with the accumulation of domesticated alleles at two seed-shattering loci, such as *sh4* and *qSH3*. Therefore, as the area of inhibition increased, the force required for detaching the seeds increased quadratically. This is comparable to a tube in which the thickness of the wall and strength of the tube increase. Awns of wild rice play a pivotal role in efficient seed dispersal when the panicles are open. The bending moment, which affects seed dispersal as a predominant factor in open panicles, was calculated. This value was found to be considerably reduced as the panicles closed. Therefore, closed panicles contribute to the stable structure of the panicles with reduced force on the spikelet base. A reduction in the bending moment and an increase in the moment of inertia of the area synergistically reduces the bending stress, enabling more seeds left in the panicles thereby contributing to rice domestication (Ishikawa *et al.* 2022).

*SPR3* is a single locus that can change panicle shape and is likely to have been visibly recognised by gatherers. In contrast, the inhibition of the abscission layer requires mutations of at least two seed-shattering loci, and the phenotypic change of partial inhibition of the abscission layer is relatively similar to that of wild rice. Therefore, closed panicles may have served as an instrumental phenotypic change that supported and enhanced the effects of *sh4* and *qSH3*.

## Common and distinct loci responsible for reducing seed-shattering degree in rice

Among the seed-shattering genes or loci that contribute to reduced seed-shattering behaviour in Asian rice, their involvement is common or specific to the groups of rice cultivars based on the genotypes of causal mutations or selective signatures in the surrounding regions. It is well known that the *sh4* mutation is conserved in all cultivated rice investigated, suggesting that the selection of *sh4* mutation may have been conducted in the early stages of rice domestication (Zhang *et al.* 2009). The closed panicle trait was also selected in the relatively early stages of rice domestication, as the cultivated rice shows a closed panicle phenotype, and the region harbouring *SPR3* experienced under selection in both *indica*, *japonica*, and aus rice cultivars (Ishikawa *et al.* 2022). In contrast, the *qSH3* mutation was conserved between *indica* and *japonica*, but absent in the circum-aus rice cultivars, suggesting that aus may have followed a separate trajectory to suppress seed shattering. Interestingly, both the *sh4* and *SPR3* regions are weakly linked on chromosome 4, implying that mutations at *sh4* and *SPR3* may have appeared in a single plant, and that the *qSH3* mutation either occurred naturally or was introduced via crossing. Therefore, circum-aus rice cultivars may have reduced the degree of seed shattering independently of *qSH3* and other loci, in addition to those selected in the *indica* and *japonica* cultivars, potentially contributing to a reduction in seed shattering. Alternatively, circum-aus cultivars carried the domesticated allele at *qSH3*, but this might have been lost due to subsequent mutations or introgression of the alleles, causing reduced seed shattering.

It has been reported that the domesticated allele at *qSH1* was only observed in some *japonica* cultivars with non-seed-shattering behaviour (Konishi *et al.* 2006, Zhang *et al.* 2009). Further analyses suggested that the *qSH1* mutation was likely selected in China during temperate *japonica* differentiation (Konishi *et al.* 2008). Additionally, the *qCSS3* region was detected as a locus contributing to the reduced seed-shattering behaviour of the *japonica* rice cultivar Npb, as the candidate region experienced a selective signature in *japonica* rice (Xu *et al.* 2012). In the analysis of *qCSS3*, there were no statistically significant peaks associated with seed shattering around the regions of *qCSS2* or *qCSS7* in the QTL-seq analysis (Sugiyama *et al.* 2023, Tsujimura *et al.* 2019), suggesting that *qCSS3* might specifically be involved in the reduced seed-shattering behaviour of *japonica* cultivars. Similarly, the *qCSS3* region was not detected as a locus associated with seed shattering in the segregating population between *indica* cultivar IR36 and wild rice *O. rufipogon*, in which *qCSS2* and *qCSS7* were detected (Sugiyama *et al.* 2023, Tsujimura *et al.* 2019), suggesting that the two loci may not be involved in the reduced seed-shattering behaviour of *japonica* cultivars and likely contributed specifically to reduced shattering of only *indica* cultivars.

These quantitative genetic studies indicate that a loss of seed-shattering behaviour during Asian rice domestication was established by mutations in the seed-shattering loci commonly conserved across the group of cultivars such as *sh4* and *qSH3* or specific to certain groups of cultivars such as *qSH1*, *qCSS2*, *qCSS3*, and *qCSS7*. Although the combination of domesticated alleles at seed-shattering loci may be the result of introgression events, such mutations at specific loci will provide important information for understanding the process of rice domestication once the causal mutations at *qCSS2*, *qCSS3*, and *qCSS7* have been identified.

## Understanding the process of rice domestication

**Fig. 3.**
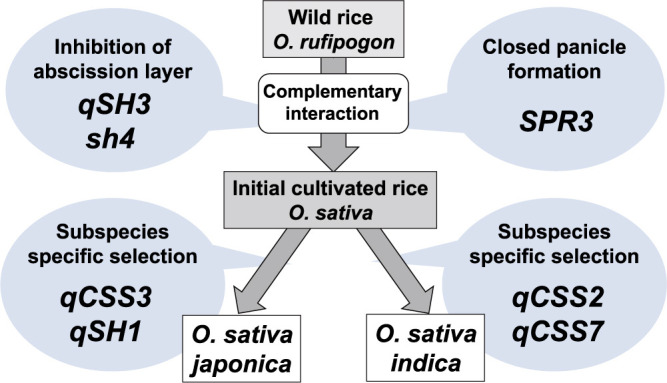
A process for rice domestication by reducing seed shattering in rice. Selections of domesticated alleles, in the early stages of rice domestication, *SPR3* causing closed panicle formation as well as *qSH3* and *sh4* cause partial inhibition of abscission layer formation. The wild rice with a slight inhibition of abscission layer and closed panicle resulted in an increase in the amount of grains left in the panicle. The subsequent selection at the subspecies-specific loci for reduced seed shattering, such as *qCSS3* and *qSH1* for *japonica* rice while *qCSS2* and *qCSS7* for *indica* rice, may have occurred to gradually increase the force required for grain detachment. These stepwise selections reducing the degree of seed shattering may be associated with the development of harvesting tools during rice domestication.

Genetic dissection of the genes or loci involved in reduced seed-shattering behaviour during rice domestication suggests that they play common and distinct roles among rice cultivars. Common factors, such as *sh4* and *qSH3* mutations, are likely to have been selected in the early stages of rice domestication, coupled with the selection of *SPR3*, a locus controlling closed panicle formation. A potential process could be stepwise selection in which wild rice with domesticated alleles of *sh4* and *SPR3* may have sequentially arisen first, as the two loci are weakly linked on chromosome 4, and then an additional mutation at *qSH3* may occur or be introgressed. Primitive wild rice with reduced seed-shattering behaviour held more seeds in the panicle, attracting ancient gatherers for harvest. The initial cultivated rice with closed panicles and reduced seed-shattering behaviour owing to the triple mutations may have been introduced elsewhere. Then, the mutations at the loci such as *qCSS3*/*qSH1* and *qCSS2*/*qCSS7* could have been selected specifically, establishing of *japonica* and *indica* as subspecies, respectively (Fig. 3). Alternatively, the domesticated alleles at these subspecies-specific seed-shattering loci may have lost because of introgression events during rice domestication.

Although there could be other possible processes that may underlie rice domestication based on population genetic studies, it is important to evaluate the plant phenotypes associated with seed shattering and gathering efficiency in the early stages of rice domestication. By employing both genomic and phenotypic studies to evaluate the selection process at domestication-related loci, rice domestication can be understood in more detail.

## Future perspectives of seed-shattering studies

Recently, rice harvesting systems have been changing, especially in Asian countries. Large-scale and machine-based harvesting systems are utilised to reduce the cost of harvesting. The cultivars grown in these countries are *indica*, which exhibits relatively easy-seed-shattering behaviour. Compared to the classical hand-harvesting system, the modern machine-based harvesting system requires fewer people and has a lower cost for employing machine operators. However, machine harvesting systems cause more seeds to shatter onto the ground during harvesting than manual harvesting, causing yield reduction and mixing of the cultivars when shattered seeds germinate in the next cropping season with other cultivars being used. Therefore, fine-tuning the degree of seed shattering to adjust it to the harvesting system is vital for future breeding. Understanding the genetic mutations which led to the reduced seed-shattering behaviour of the current rice cultivars is essential for maximising yield. The new genetic modifications may appear differently due to the potential mutations in the genetic background of cultivars. In addition, subspecies-specific loci for reduced seed-shattering behaviour can be replaced by functional alleles when cultivars across subspecies are crossed to breed novel cultivars. Mutations at the common loci, such as *sh4* and *qSH3*, in *indica* and *japonica*, cause a slight inhibition of the abscission layer in wild rice, but if other functional alleles at subspecies-specific seed-shattering loci are accumulated in a hybridised offspring, a few of them may acquire seed-shattering ability based on Mendelian inheritance. Once plants obtain seed-shattering abilities, they may propagate themselves, which may act as a step towards weedy rice. Therefore, identifying the genes or loci contributing to reduced seed-shattering behaviour in the present cultivars is essential to minimising side effects of weediness, which may serve as the basis of crop feralisation or de-domestication due to the multiple selections for present cultivars.

As mentioned in the introduction section, the process and chronology of rice domestication have not been clarified, as the plants in the process do not exist today. However, studies using DNA from excavated samples are increasing, ancient rice DNA will provides useful information regarding the chronology of selection. Evaluation of these genotypes, as well as the generation of ILs with domestication-related traits, is more vital to clarifying the process of rice domestication.

## Author Contribution Statement

RI conceptualised the review and wrote the manuscript.

## Figures and Tables

**Table 1.** Loci involved in a reduction in seed shattering during Asian rice domestication

Loci	Encoded protein	RAP (Os ID)	MSU (LOC_Os ID)	References	Causal mutation
*sh4*/*SHA*	Trihelix transcription factor	Os04g0670900	LOC_Os04g57530	Li *et al.* (2006b), Lin *et al.* (2007)	SNP
*qSH1*	BEL1-type homeobox protein	Os01g0848400	LOC_Os01g62920	Konishi *et al.* (2006)	SNP^1)^
*qSH3*	YABBY transcription factor	Os03g0650000	LOC_Os03g44710	Ishikawa *et al.* (2022)	SNP
*qCSS3*	–	–	–	Tsujimura *et al.* (2019)	–
*qCSS2*	–	–	–	Sugiyama *et al.* (2023)	–
*qCSS7*	–	–	–	Sugiyama *et al.* (2023)	–

^1)^ Causal mutation is an SNP controlling downstream gene.

**Table 2.** Genes controlling seed shattering identified by artificially induced mutagenesis or reverse genetic approaches in rice

Gene	Encoded protein	RAP (Os ID)	MSU (LOC_Os ID)	References
*OsCPL1*	carboxy-terminal domain (CTD) phosphatase	Os07g0207700	LOC_Os07g10690	Ji *et al.* (2010)
*SHAT1*	APETALA2 transcription factor	Os04g0649100	LOC_Os04g55560	Zhou *et al.* (2012)
*SNB*	APETALA2-like transcription factor	Os07g0235800	LOC_Os07g13170	Jiang *et al.* (2019)
*Sh13*	osa-mir172d	–	–	Li *et al.* (2019)
*OsSh1* ^1)^	YABBY transcription factor	Os03g0650000	LOC_Os03g44710	Li *et al.* (2020), Lin *et al.* (2012)
*SH5*	BEL1-type homeobox protein	Os05g0455200	LOC_Os05g38120	Yoon *et al.* (2014)
*OSH15*	KNOX protein	Os07g0129700	LOC_Os07g03770	Yoon *et al.* (2017)

^1)^ *OsSh1* is the gene encoded by *qSH3*.

**Table 3.** Seed-shattering loci or genes identified based on the studies of African rice, *O. glaberrima*

Loci	Encoded protein	Gene code in *O. sativa* (RAP)	References	Causal mutation
*SH3*	YABBY transcription factor	Os03g0650000	Lv *et al.* (2018), Wang *et al.* (2014)	Whole deletion
*SH4*/*SH3*^1)^/*GL4*	Trihelix transcription factor	Os04g0670900	Win *et al.* (2017), Wu *et al.* (2017)	SNP
*SH5*	BEL1-type homeobox protein	Os05g0455200	Cubry *et al.* (2018)	Deletion^2)^
*SH11* ^3)^	MYB transcription factor	Os11g0684000	Ning *et al.* (2023)	N.A.^4)^

^1)^ Name of *SH3* is corresponding to the first report of the locus by Eiguchi and Sano (1990), later the locus was named as *sh4* (Li *et al.* 2006a). ^2)^ Effect of the mutation at *SH5* (a deletion in the coding region) on the degree of seed shattering need to be elucidated in *O. glaberrima*. ^3)^ The locus was identified based on the analysis of seed-shattering behaviour of the introgression line obtained between *O. glaberrima* and *O. sativa*. ^4)^ Causal mutation for *SH11* is not clearly known, but *O. glaberrima* and *O. sativa* likely encode functional and non-functional alleles, respectively.
